# Supplementary figures and images for: Endogenous Syngap1 alpha splice forms promote cognitive function and seizure protection
Source: eLife. 2022 Apr 8;11:e75707. doi: 10.7554/eLife.75707 (PMC9064290; doi:10.7554/eLife.75707)

Fig 2 - source data 1

Panel C

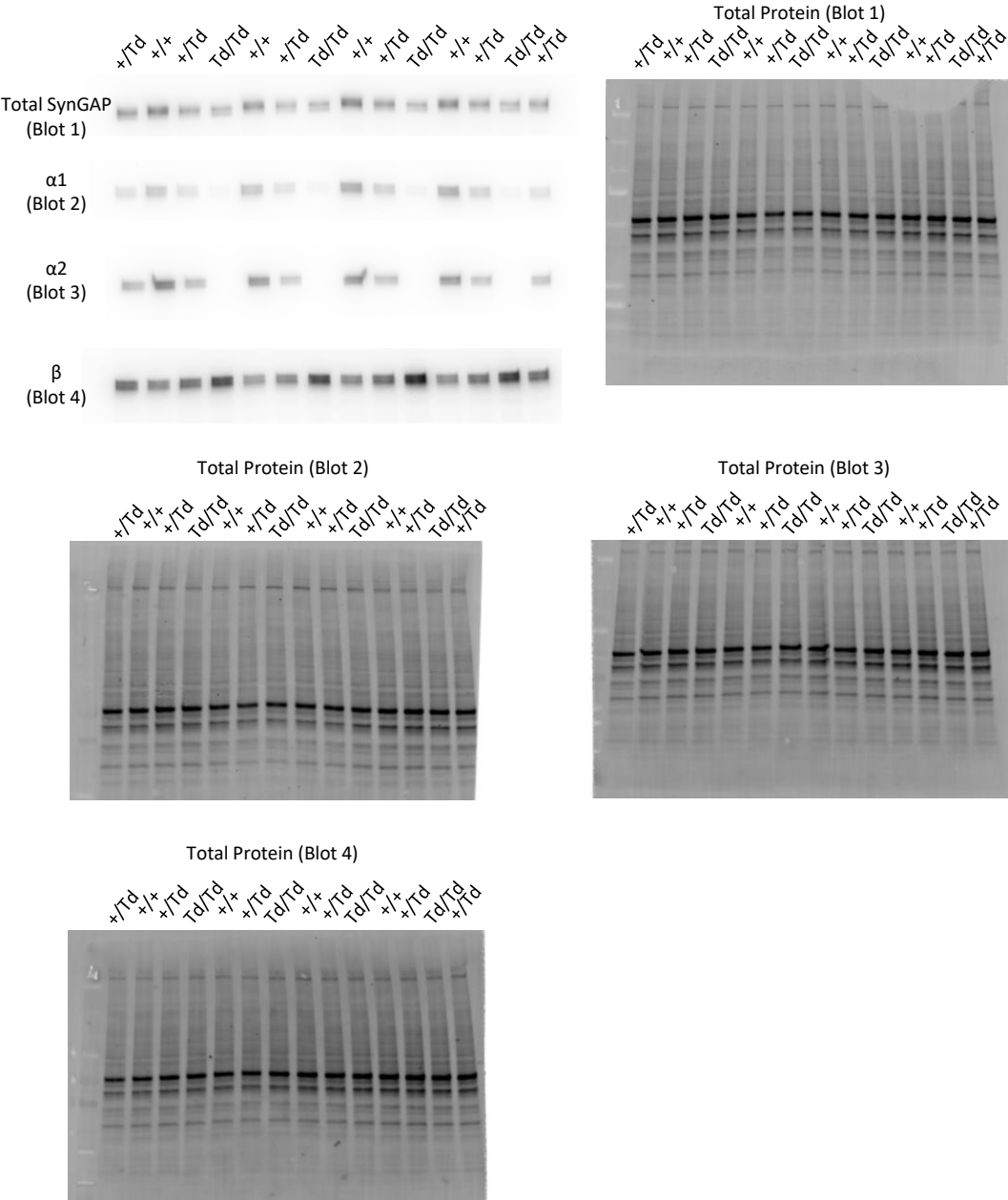

Supplement: Figure 2—source data 1. [file elife-75707-fig2-data1.pdf]

Fig 2 Supplement - source data 2

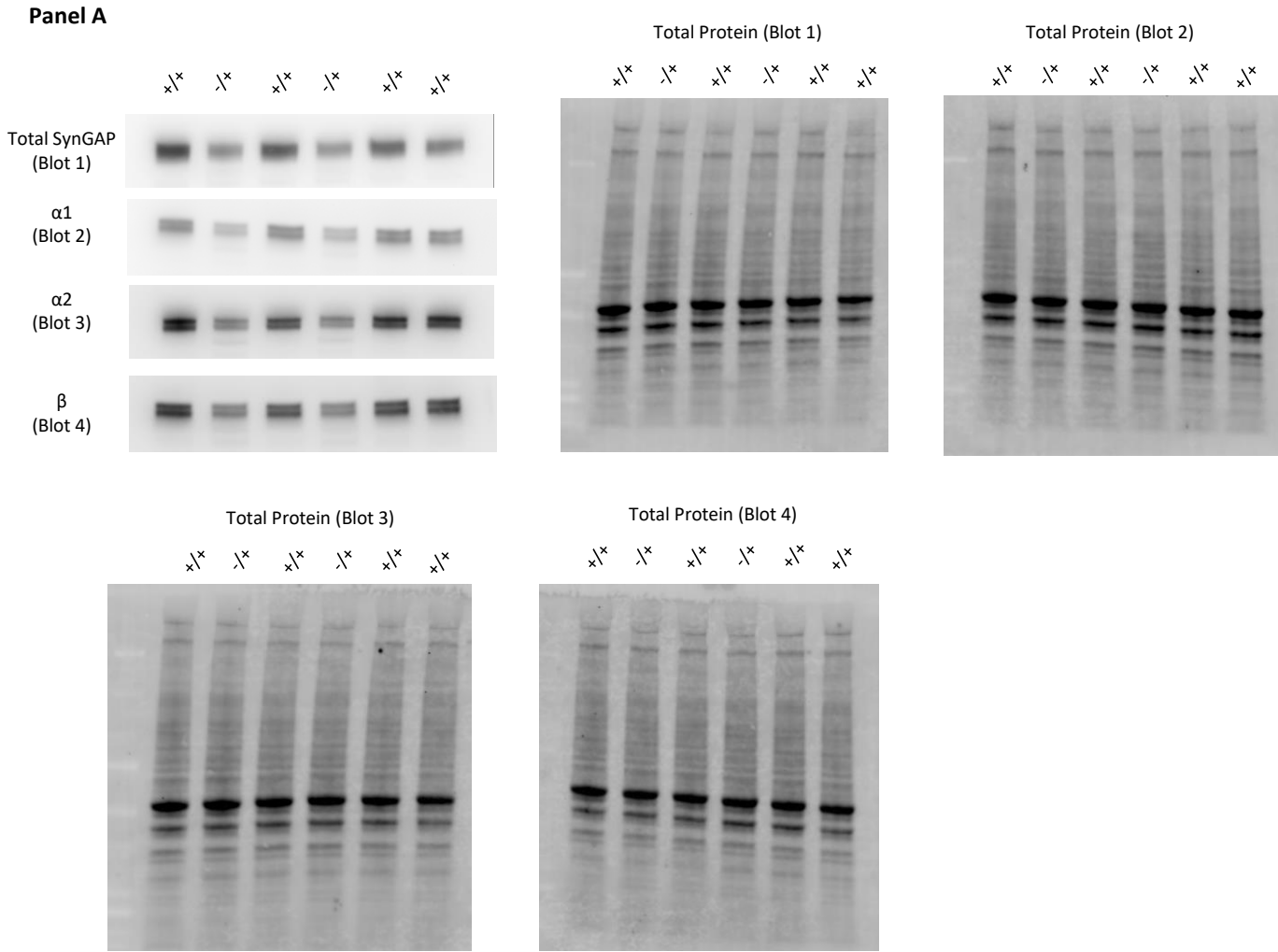

Fig 2 Supplement - source data 2

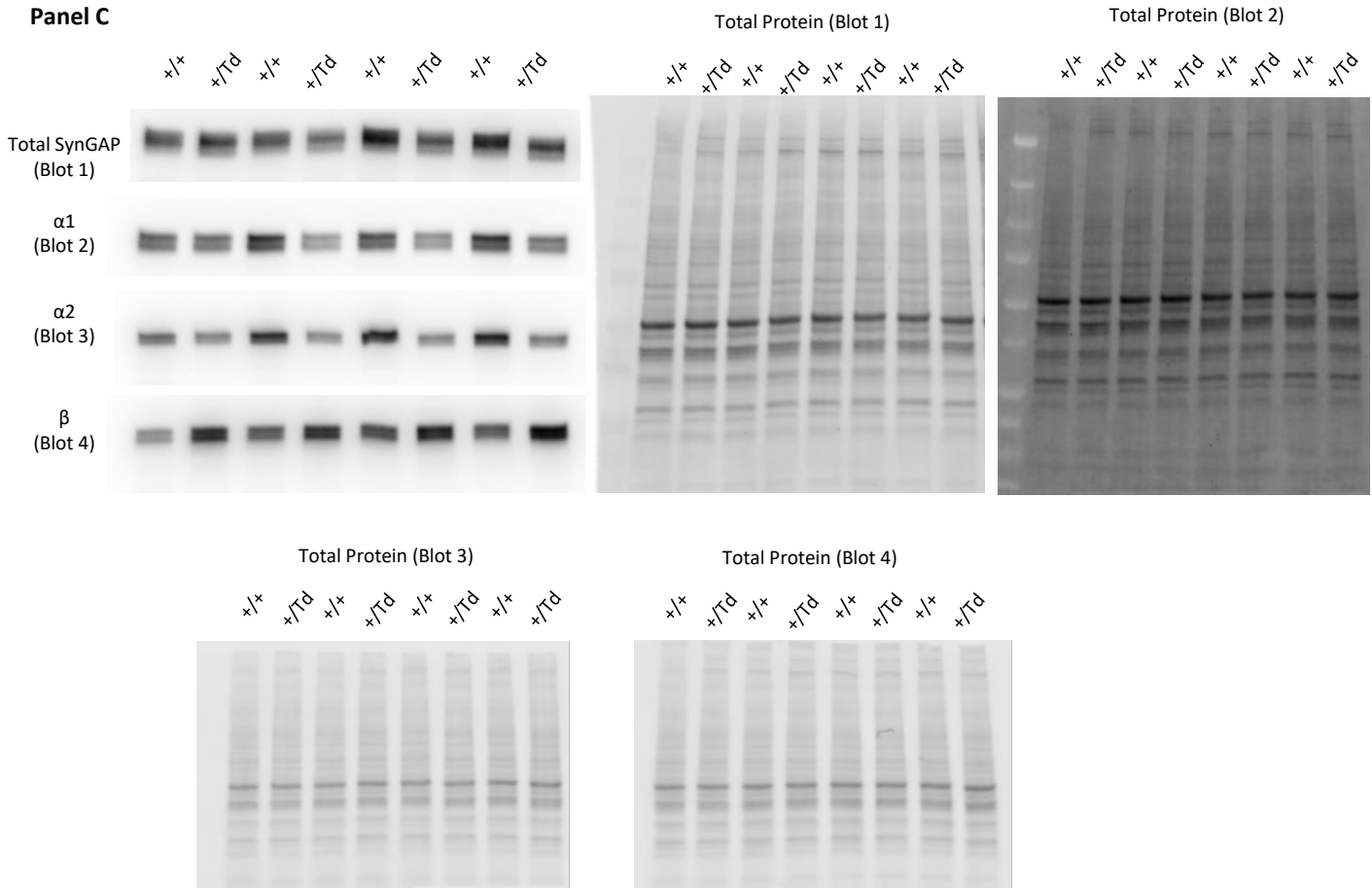

Supplement: Figure 2—figure supplement 1—source data 1. [file elife-75707-fig2-figsupp1-data1.pdf]

Fig 3 - source data 3

Panel C

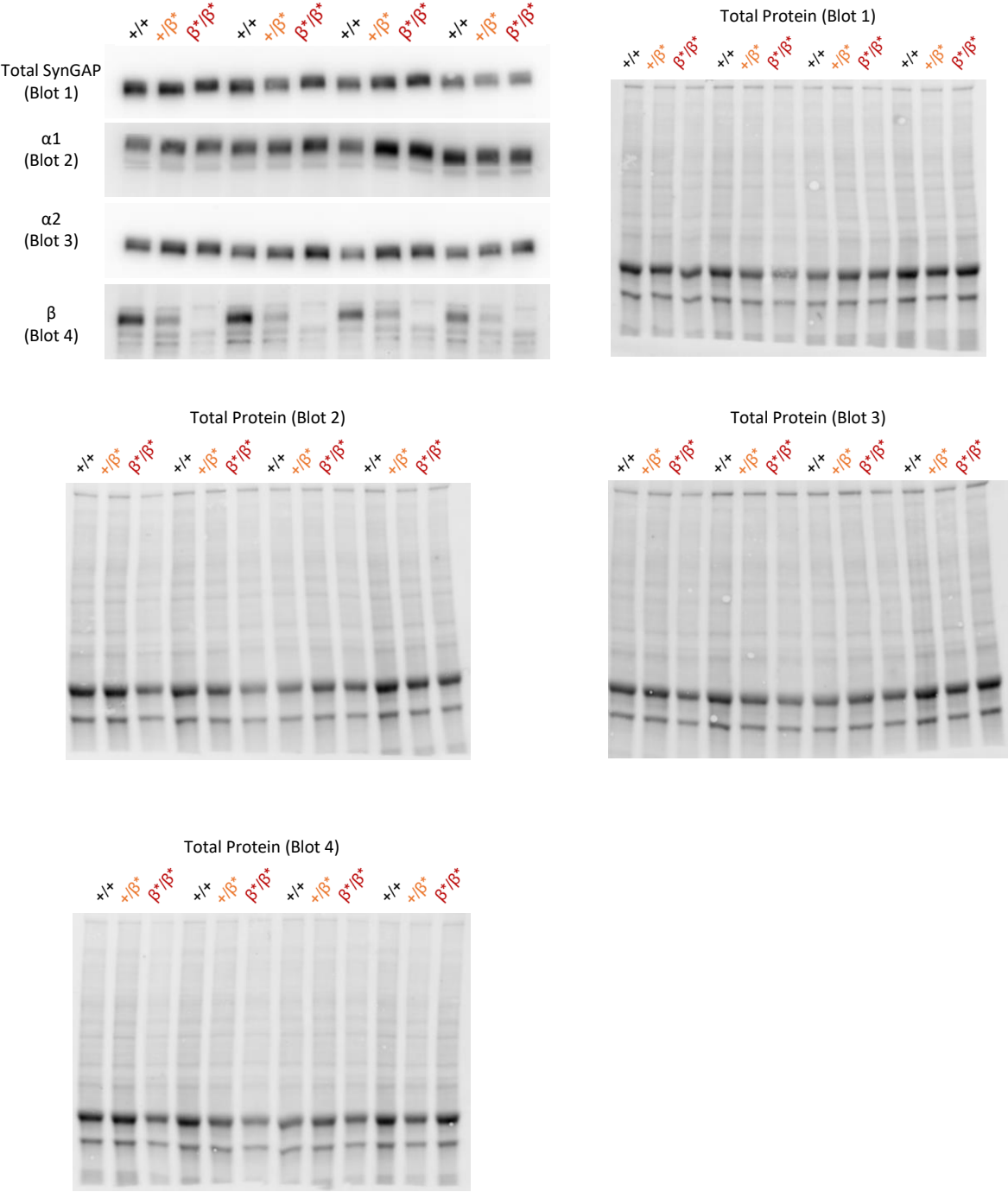

Supplement: Figure 3—source data 1. [file elife-75707-fig3-data1.pdf]

Fig 3 Supplement - source data 4

Panel A

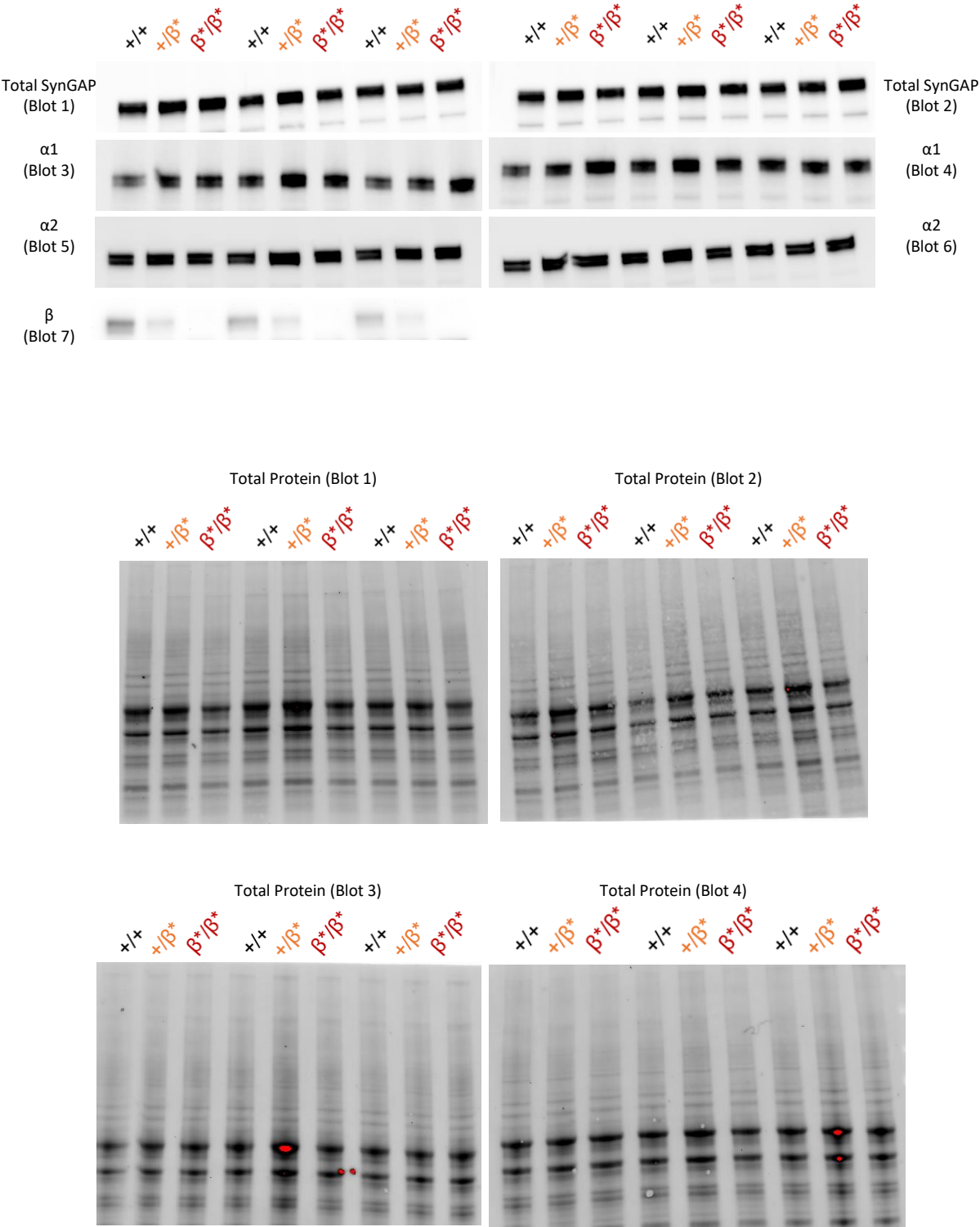

Fig 3 Supplement - source data 4

Panel A

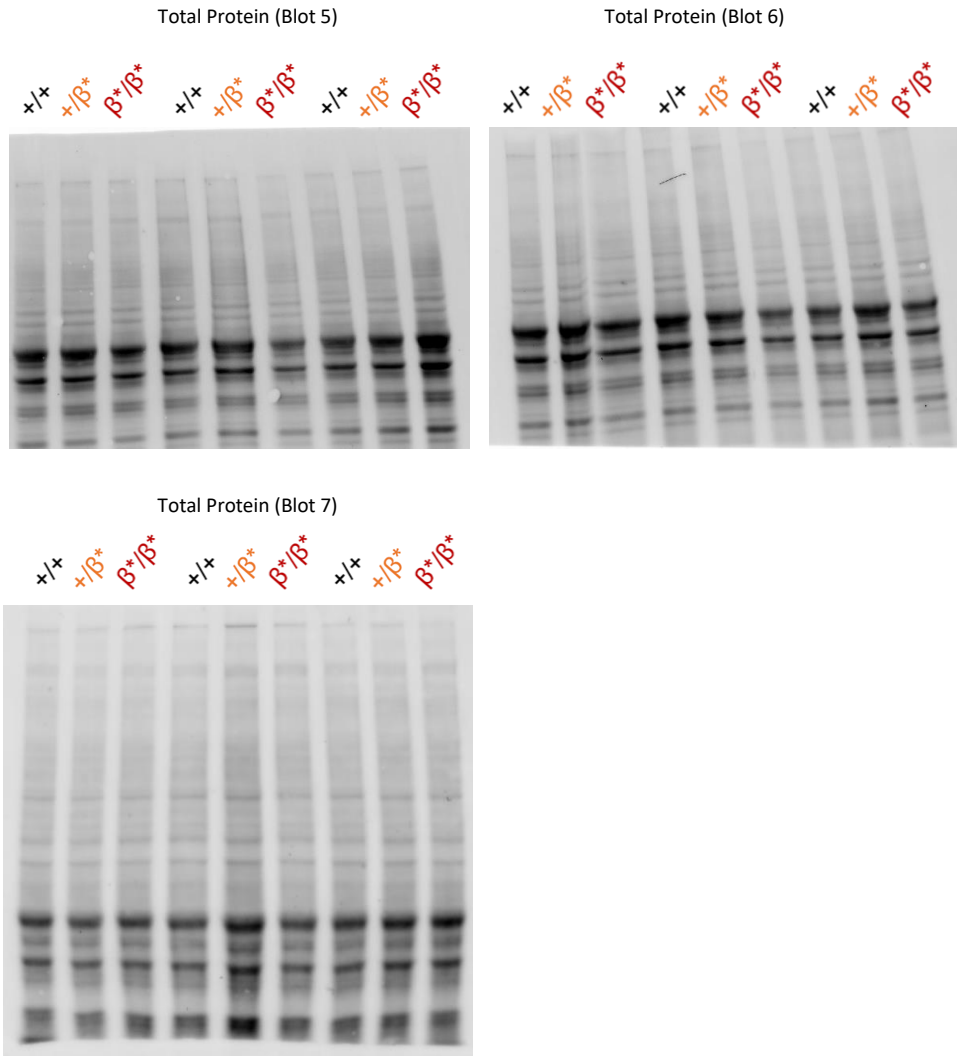

Supplement: Figure 3—figure supplement 1—source data 1. [file elife-75707-fig3-figsupp1-data1.pdf]

Fig 4 - source data 5

Panel B

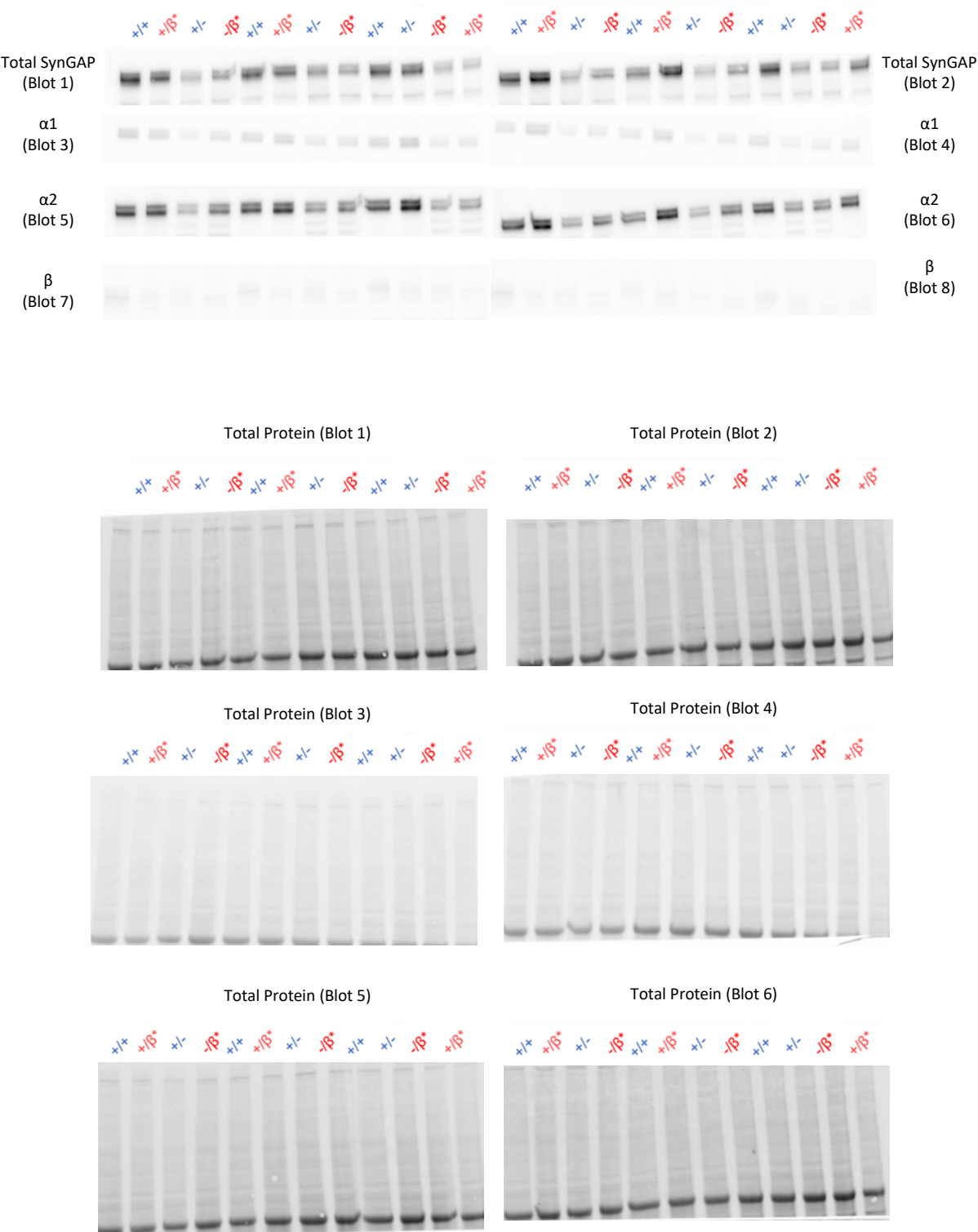

Fig 4 - source data 5

Panel B

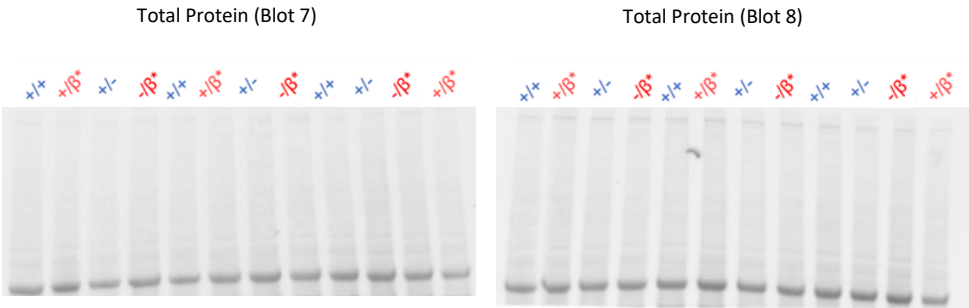

Supplement: Figure 4—source data 1. [file elife-75707-fig4-data1.pdf]

Fig 4 Supplement - source data 6

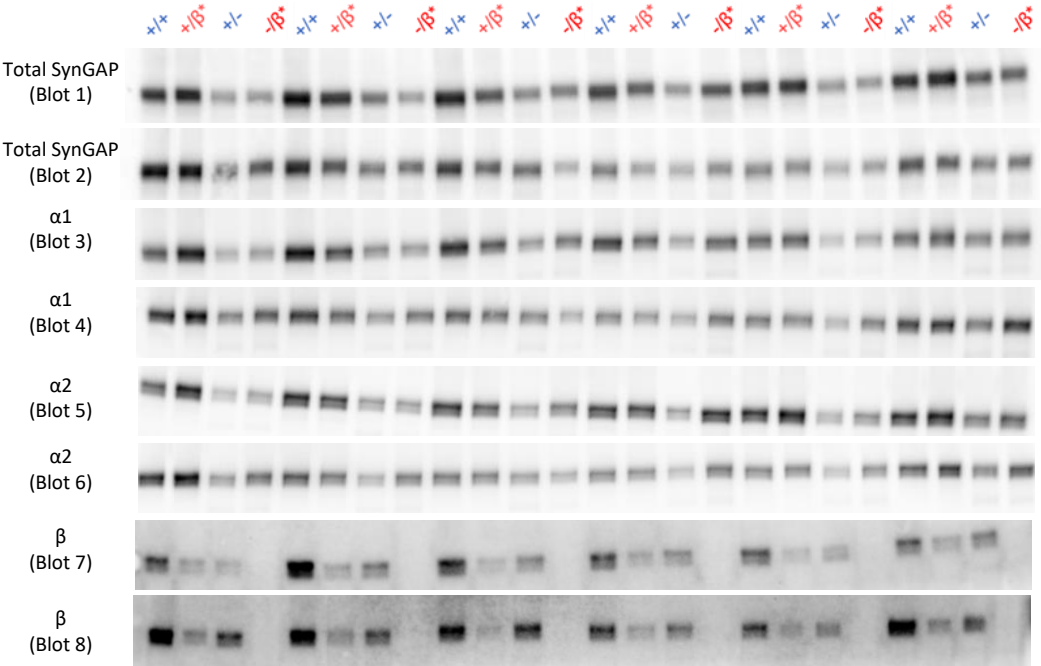

Total Protein (Blot 1)

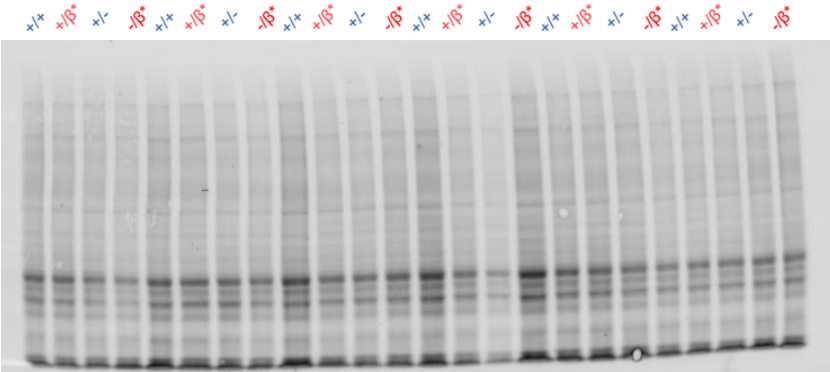

Total Protein (Blot 2)

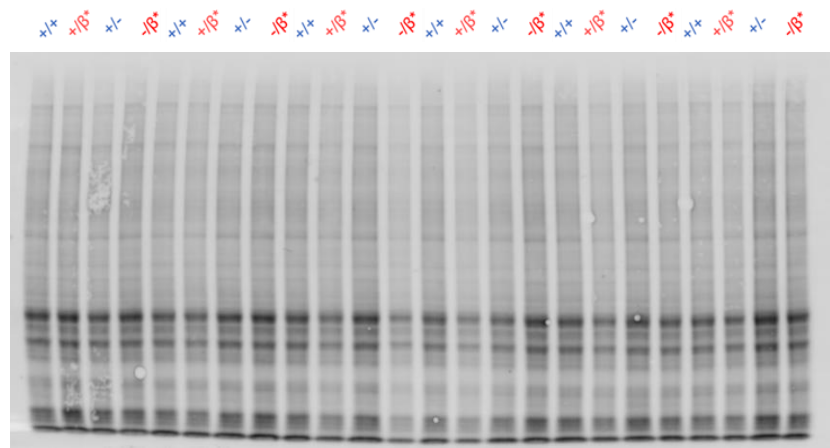

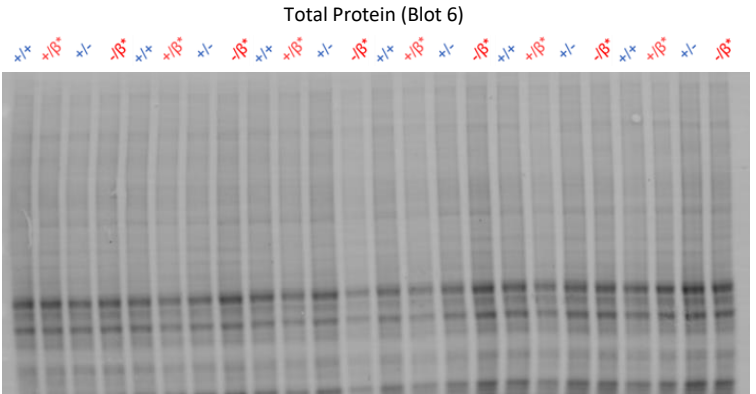

Fig 4 Supplement - source data 6

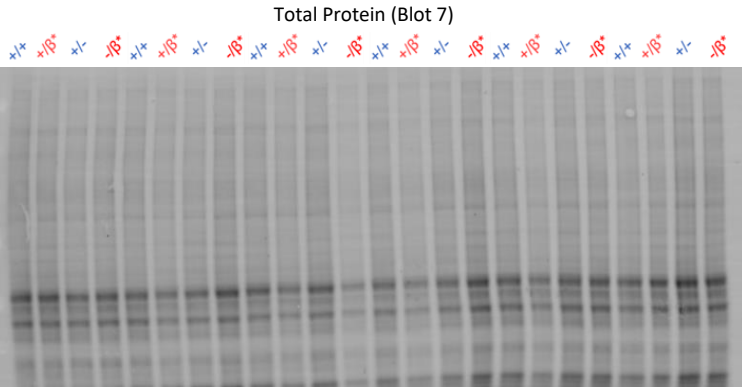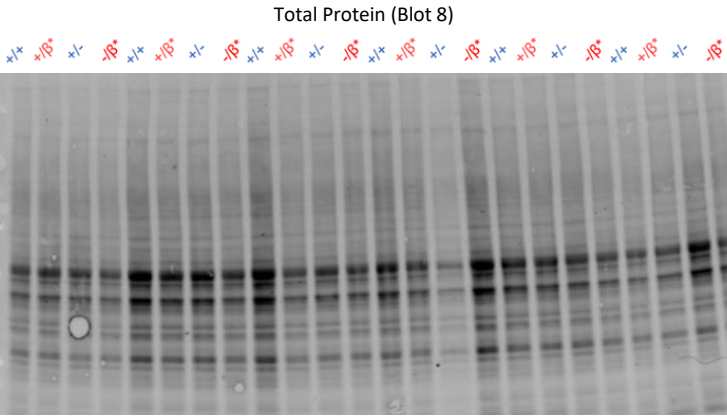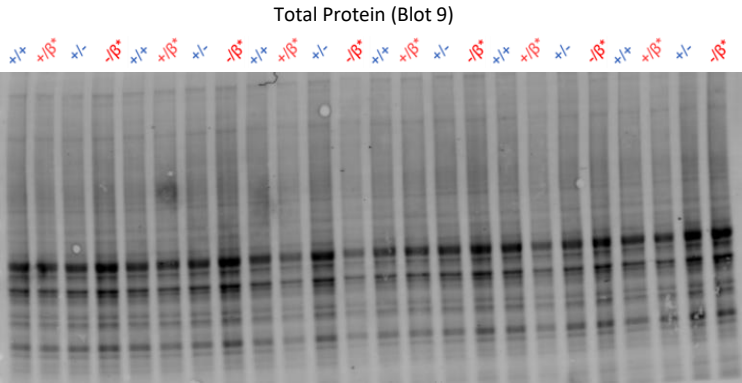

Supplement: Figure 4—figure supplement 1—source data 1. [file elife-75707-fig4-figsupp1-data1.pdf]

**Fig 5 - source data 7**

### Panel I

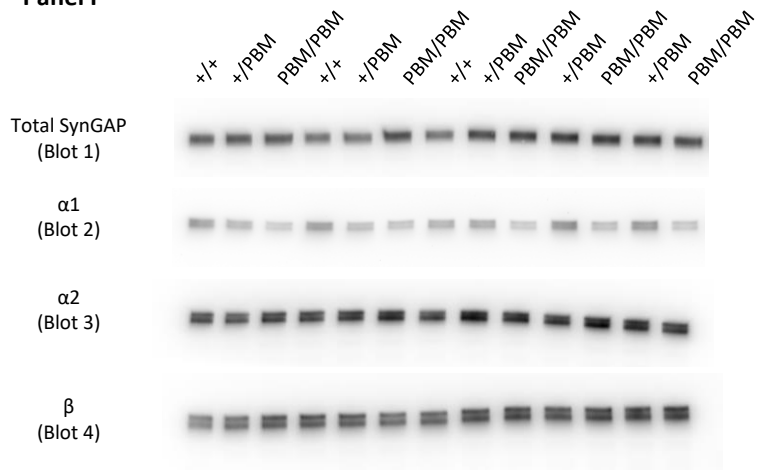

Supplement: Figure 5—source data 1. [file elife-75707-fig5-data1.pdf]

Fig 5 Supplement - source data 8

Panel B

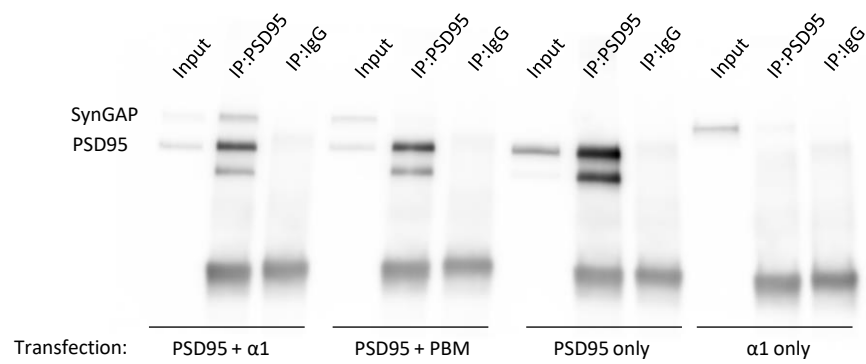

Panel H

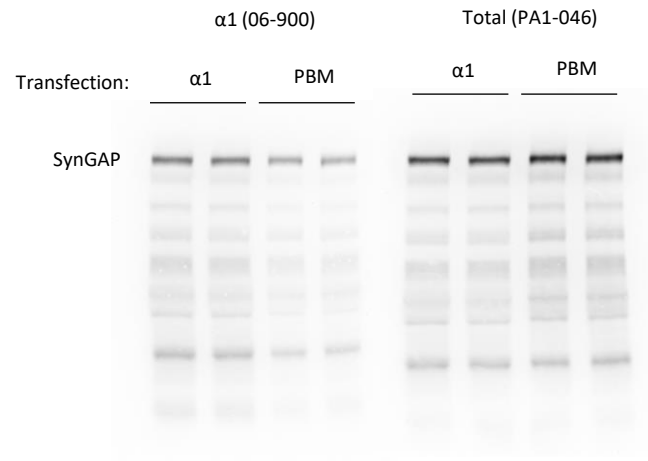

Supplement: Figure 5—figure supplement 1—source data 1. [file elife-75707-fig5-figsupp1-data1.pdf]

Fig 6 - source data 9

Panel A

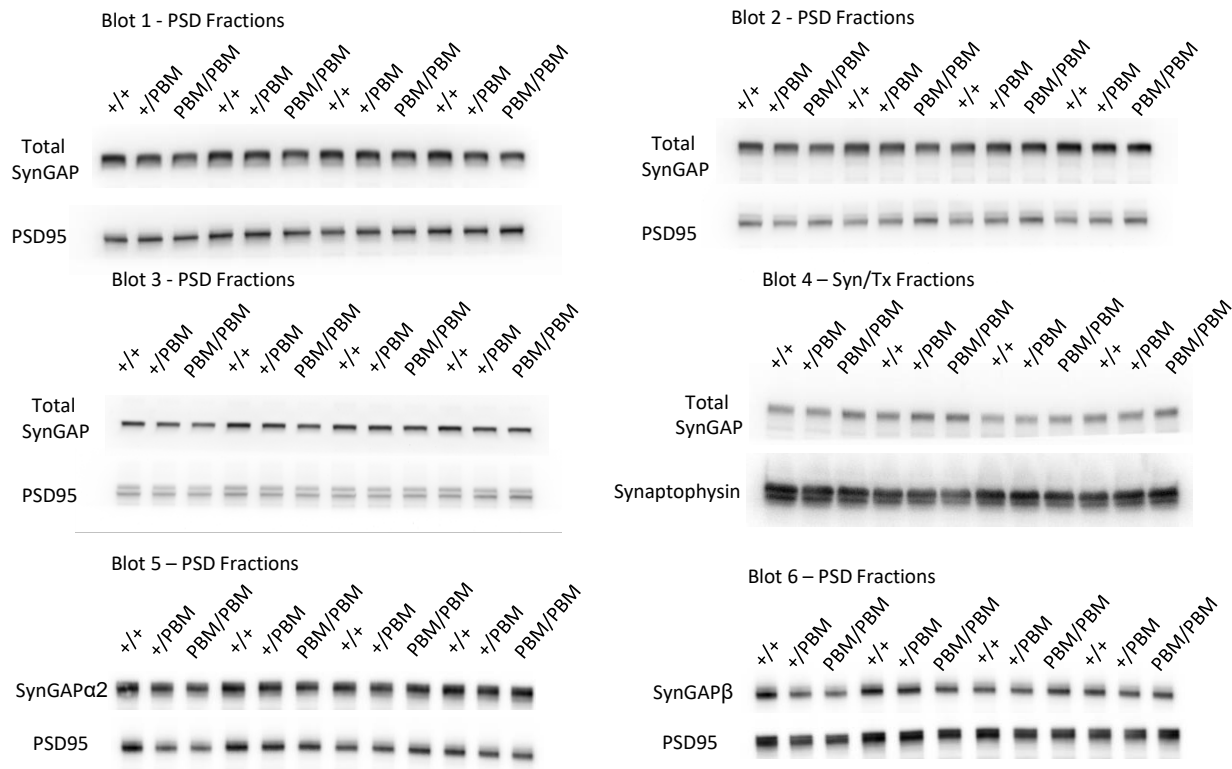

Panel B

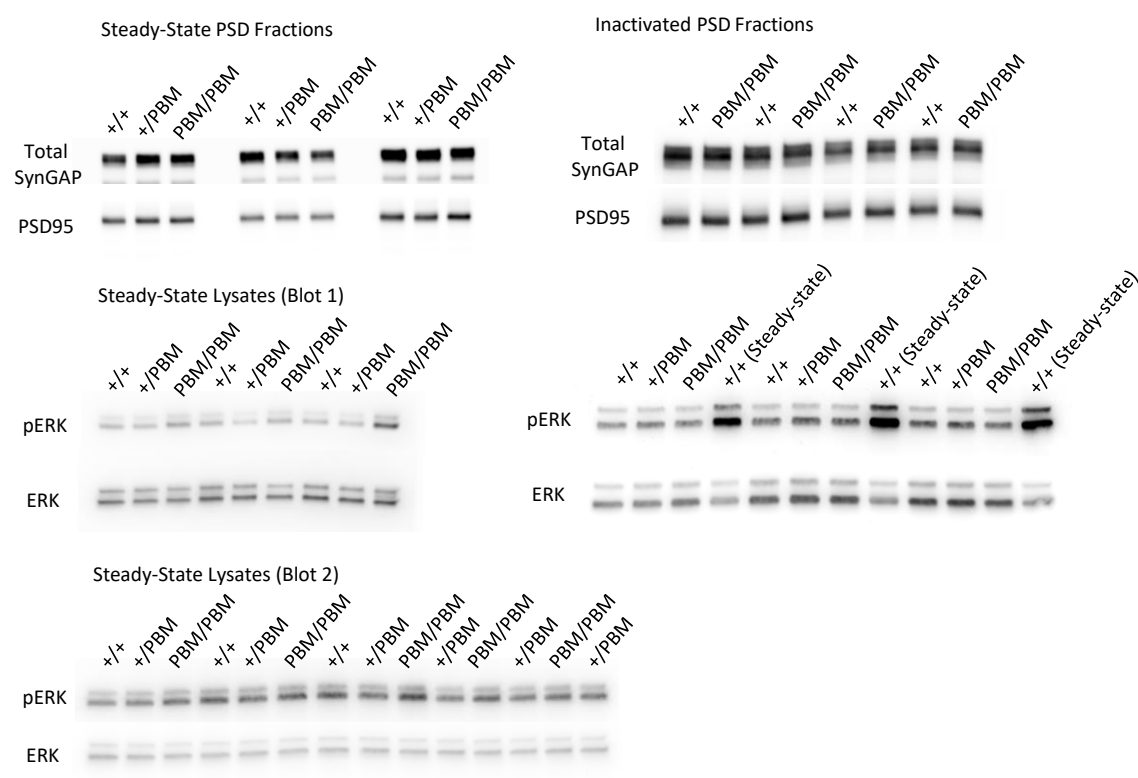

Supplement: Figure 6—source data 1. [file elife-75707-fig6-data1.pdf]

Fig 6 Supplement - source data 10

Panel A

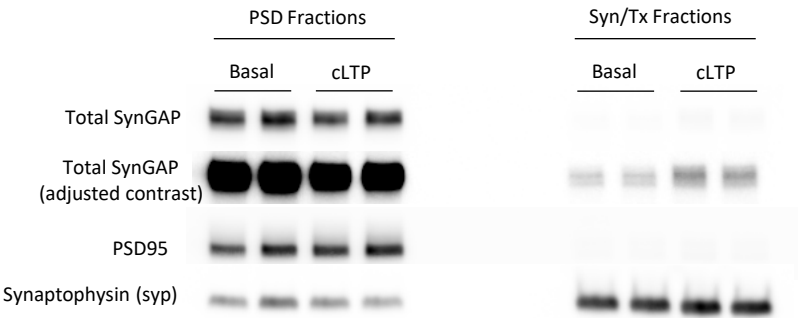

Panel B

Blot 1

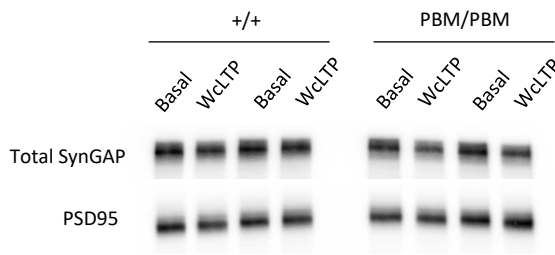

Blot 2

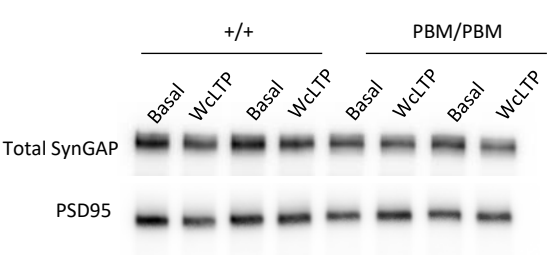

Blot 3

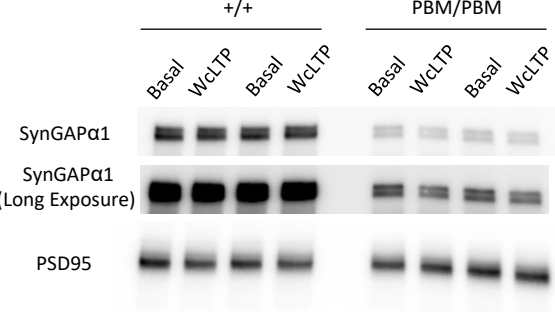

Blot 4

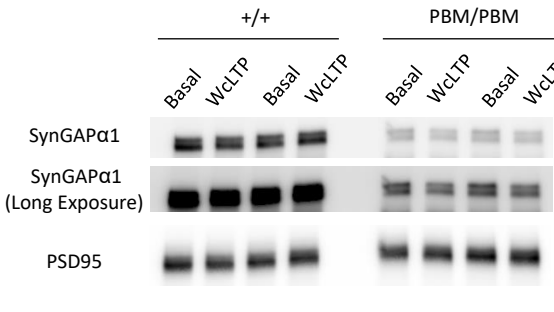

Blot 5

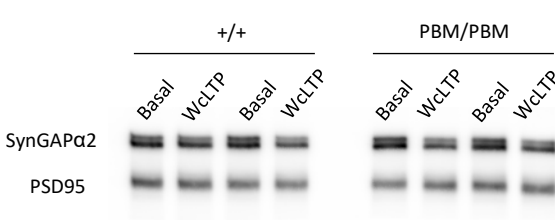

Blot 6

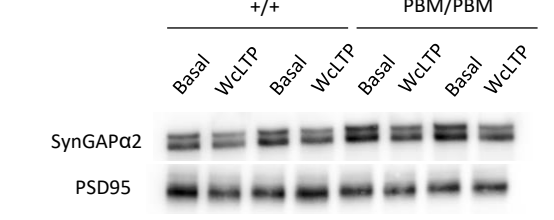

Blot 7

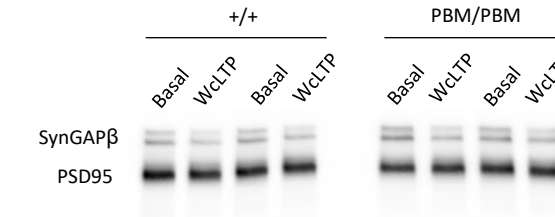

Blot 8

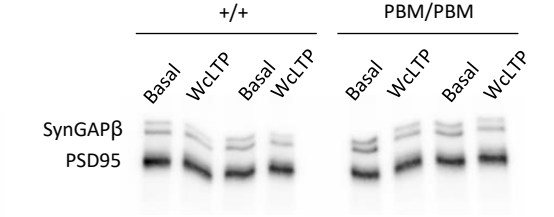

Supplement: Figure 6—figure supplement 1—source data 1. [file elife-75707-fig6-figsupp1-data1.pdf]

**Fig 7 - source data 11**

### Panel D

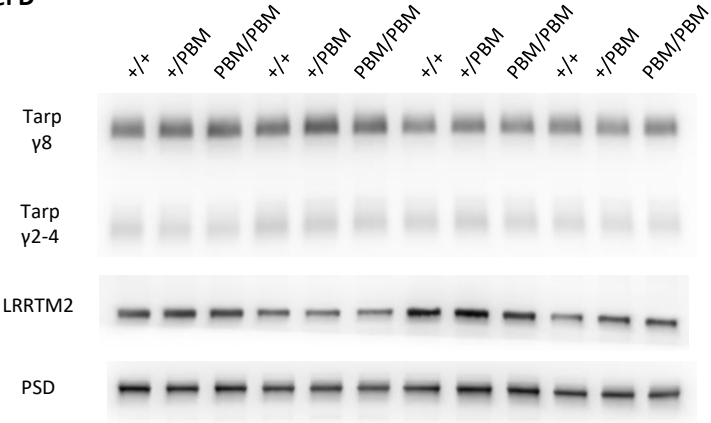

Supplement: Figure 7—source data 2. [file elife-75707-fig7-data2.pdf]
