# Supplementary material for: Endogenous Syngap1 alpha splice forms promote cognitive function and seizure protection
Source: eLife. 2022 Apr 8;11:e75707. doi: 10.7554/eLife.75707 (PMC9064290; doi:10.7554/eLife.75707)
Supplement: Supplementary file 1. [file elife-75707-supp1.docx]

**Supplementary File 1**

| **Protein** | **+/+ vs** | | | | | **β* Allele Effect**  **in *Syngap1* Hets** | |
| --- | --- | --- | --- | --- | --- | --- | --- |
|  | **+/-** | **+/Td** | **Td/Td** | **β*/β*** | **PBM/PBM** | **+/β*** *($***)** | **-/β*** *(#)* |
| **Total SynGAP** | ~50% | ~25%  ↓↓ | ~50%  ↓↓↓ | ⇔ | ⇔ | ⇔ | ~50%  ↓↓↓ |
|  | ↓↓ |  |  |  |  |  |  |
| **SynGAP-α1** | ~50% | ~50% | ~95%  ↓↓↓ | Increased | ⇔ | ~10-20% | 50% |
|  | ↓↓ | ↓ |  | ↑ | (*) | ↑↑↑ | ↑↑↑ |
| **SynGAP-α2** | ~50% | ~50% | ~95%  ↓↓↓ | Increased | ⇔ | ~10-20% | ~50% |
|  | ↓ | ↓↓ |  | ↑ |  | ↑↑↑ | ↑↑↑ |
| **SynGAP-β** | ~50% | ~50% | ~100%  ↑↑ | ~0% | ⇔ | ~50% | ~0% |
|  | ↓ | ↑↑ |  | ↓↓↓ |  | ↓↓↓ | ↓↓↓ |
|  | **Fig.S2** | **Fig. 2/S2** |  | **Fig. 3 and S3** | **Fig. 5** | **Fig. 4 and S4** | |
|  |  |  |  |  |  | |  |
|  |  |  |  |  |  |  |  |

| **Behavior** | **+/+ vs** | | | | **β* Allele Effect**  **in *Syngap1* Hets** | | |  |  |
| --- | --- | --- | --- | --- | --- | --- | --- | --- | --- |
|  | **+/Td** | **Td/Td** | **β*/β*** | **PBM/PBM** | **+/β*** *($)* | **-/β*** *(#)* | **+/-** *($)* |  |  |
| **Survival** | ⇔ | Reduced | ⇔ | ⇔ | ⇔ | ⇔ | ⇔ |  |  |
|  |  | ↓↓↓ |  |  |  |  |  |  |  |
| **Locomotion** | Elevated | Elevated | Reduced | Elevated | Reduced | Reduced | Elevated |  |  |
|  | ↑↑↑ | ↑↑↑ | ↓↓ | ↑↑↑ | ↓ | ↓ | ↑↑↑ |  |  |
| **Seizure Threshold** | More sensitive | *N/A* | Protection | More sensitive | Protection | Protection | More sensitive |  |  |
|  | ↓↓↓ |  | ↑↑↑ | ↓↓ | ↑ | ↑ | ↓↓↓ |  |  |
| **Remote Memory (FC)** | Impaired  Memory | *N/A* | ⇔ | Impaired  Memory | ⇔ | ⇔ | Impaired  Memory  ↓↓↓ |  |  |
|  | ↓ |  |  | ↓↓↓ |  |  |  |  |  |
| **Morris Water Maze** | ⇔ | *N/A* | Enhanced  Learning | Impaired  Learning | *N/A* | *N/A* | *N/A* |  |  |
|  |  |  | ↑↑ | ↓↓↓ |  |  |  |  |  |
|  | **Fig. 2** | | **Fig. 3** | **Fig. 8** | **Fig. 4** | | |  | **Previously published** |

[continued on next page]

| **E-Phys** | **+/+ vs** | | |
| --- | --- | --- | --- |
|  | **+/Td** | **β*/β*** | **PBM/PBM** |
| **LTP** | *N/A* | *N/A* | Impaired |
|  |  |  | ↓↓↓ |
| ***m*EPSC Amplitude** | Increased | Reduced | Increased |
|  | ↑↑↑ | ↓↓ | ↑↑↑ |
| ***m*EPSC Frequency** | Increased | Reduced | Increased |
|  | ↑↑↑ | ↓↓↓ | ↑↑↑ |
|  | **Fig. 9** | | |

| ⇔ = no change |
| --- |
| ↓ = *p <0.05* |
| ↓↓ = *p <0.01* |
| ↓↓↓ = *p <0.001* |
| ↑ = *p <0.05* |
| ↑↑ = *p <0.01* |
| ↑↑↑ = *p <0.001* |

***($*)** relative to +/+

***(#*)** relative to +/-

**(*)** Reduced antibody affinity
